# Quantitative assessment of early biomechanical modifications in diabetic foot patients: the role of foot kinematics and step width

**DOI:** 10.1186/s12984-015-0093-6

**Published:** 2015-11-09

**Authors:** Giuseppe Lamola, Martina Venturi, Dario Martelli, Elisabetta Iacopi, Chiara Fanciullacci, Alberto Coppelli, Bruno Rossi, Alberto Piaggesi, Carmelo Chisari

**Affiliations:** Unit of Neurorehabilitation, Department of Neuroscience, University Hospital of Pisa, Via Paradisa 2, 56124 Pisa, Italy; The BioRobotics Institute, Scuola Superiore Sant’Anna, Pontedera, Pisa, viale Rinaldo Piaggio 34, 56025 Pisa, Italy; Diabetic Foot Section, Department of Medicine, University Hospital of Pisa, Via Paradisa 2, 56124 Pisa, Italy

**Keywords:** Type 2 diabetes mellitus, Foot ulcer, Diabetic neuropathy, Gait analysis

## Abstract

**Background:**

Forefoot ulcers (FU) are one of the most disabling and relevant chronic complications of diabetes mellitus (DM). In recent years there is emerging awareness that a better understanding of the biomechanical factors underlying the diabetic ulcer could lead to improve the management of the disease, with significant socio-economic impacts. Our purpose was to try to detect early biomechanical factors associated with disease progression.

**Methods:**

Thirty subjects (M/F: 22/8; mean age ± SD: 61,84 ± 10 years) with diagnosis of type II DM were included. The participants were divided into 3 groups (10 subjects per group) according to the stage of evolution of the disease: Group 1, subjects with newly diagnosed type II DM, without clinical or instrumental diabetic peripheral neuropathy (DPN) nor FU (group called “DM”); Group 2, with DPN but without FU (group called “DPN”); Group 3, with DPN and FU (group called “DNU”). All subjects underwent 3-D Gait Analysis during walking at self-selected speed, measuring spatio-temporal, kinematic and kinetic parameters and focusing on ankle and foot joints. The comparative analysis of values between groups was performed using 1-way ANOVA. We also investigated group to group differences with Tukey HSD test. The results taken into consideration were those with a significance of *P* < 0,05. 95 % confidence interval was also calculated.

**Results:**

A progressive and significant trend of reduction of ROM in flexion-extension of the metatarso-phalangeal joint (*P* = 0.0038) and increasing of step width (*P* = 0.0265) with the advance of the disease was evident, with a statistically significant difference comparing subjects with recently diagnosed diabetes mellitus and subjects with diabetic neuropathy and foot ulcer (*P* = 0.0048 for ROM and *P* = 0.0248 for step width at Tukey’s test).

**Conclusions:**

The results provide evidence that foot segmental kinematics, along with step width, can be proposed as simple and clear indicators of disease progression. This can be the starting point for planning more targeted strategies to prevent the occurrence and the recurrence of a FU in diabetic subjects.

## Background

Forefoot ulcers (FU) are one of the most disabling and relevant chronic complications of diabetes mellitus (DM) and, among DM patients, the lifetime risk of developing a FU is estimated to be 15 to 20 % [1]. Besides, FU are frequently cause of hospitalization, but also of amputation and mortality in DM patients: between 40 and 70 % of all the amputations of the lower extremities is related to DM and about 85 % of diabetes-related lower extremity amputations is a result of FU [2].

In recent years there is emerging awareness that a better understanding of the biomechanical factors underlying the diabetic ulcer could lead to improve the management of the problem, with significant socio-economic implications. A clear relationship between ulcer and biomechanics is yet to be fully specified, even if it seems important to focus attention on alterations due to peripheral neuropathy.

Peripheral neuropathy is one of the key factors in the pathogenesis of diabetic foot. Typically, it is a sensorimotor, symmetric and distal neuropathy and may include symptoms like pain and autonomic dysfunctions [3]. From the point of view of posture and gait, diabetic peripheral neuropathy (DPN) determines alterations by itself, with the contribution of both sensory and motor deficits [4, 5].

Previous studies found that gait characteristics in diabetic neuropathic subjects may be described as a conservative gait pattern [6] characterized by a reduction of walking speed and cadence (step/min), a prolonged duration of stance phase, an increased time of double support and a greater step-to-step variability [7]. This is consistent with a progressive reduction in muscle strength in patients with overt polyneuropathy. The muscles involved are particularly ankle plantar and dorsiflexors [8, 9], but also in a lesser degree knee flexors and extensors [4]. Tuttle et al. [10] showed in obese subjects with DM and DPN an increased intermuscular adipose tissue of the triceps surae (measured with non-invasive MRI) associated with reduced muscle strength, related to reduced power of ankle plantar and dorsiflexion (quantified using the isokinetic dynamometer) and to impaired physical performance.

It is thought that peripheral neuropathy combines with factors such as such as elevated plantar pressures as a result of tissue changes and deformity for outlining a biomechanical pathway to ulceration. An extensive body of work is dedicated to plantar pressure measurement as a biomechanical factor for predicting ulcers. Rao et al. [11] examined the mechanisms contributing to sustained plantar loading during gait, finding an increase in plantar loading in patients with DM, compared to the control group, associated to modifications in segmental foot mobility. There is also significant emerging evidence that incorporating plantar pressure measurements into intervention design can improve clinical outcomes. Pressure reduction in footwear is likely to have beneficial effects, so one factor in a multidimensional strategy to prevent ulcer recurrence.

A crucial question is the occurrence of biomechanical modifications of gait pattern over the time course of the disease. At the time of DM diagnosis, about 10–18 % of patients appears to be already affected by peripheral neuropathy, as demonstrated by studies on motor and sensory conduction velocity [12]; it has been shown that some alterations of gait become evident in those subjects even in the absence of clinical evidences of peripheral neuropathy [5, 13]. Moreover, plantar pressure distribution patterns in prediabetes subjects studied with baropodometer showed similar alterations to that often found in DM subjects [14]. Therefore, early detection of peculiar gait pattern appears to be fundamental in order to improve the long-term management and prevention of ulcers and their frequent relapses.

On the other hand, to date only few studies included patients with a current or past history of diabetic FU among the patients assessed with gait analysis [15–18]: this is probably due to the greater difficulty of exams execution and data standardization in these patients. Anyway, their evaluation can help defining an evolutionary path that begins from subjects with newly diagnosed type II DM (which does not show any clinical feature of peripheral neuropathy) and ends with the FU.

Until now biomechanical factors in diabetic patients have been described in a dispersive manner, without writing up a clear summary focused on single aspects on which to intervene. In this context, an element that would help in the treatment is to find a single (or a short series of) biomechanical factor able to reveal in early stages that a diabetic patient will have a higher probability to develop a FU, in order to help planning early and more targeted strategies to prevent FU.

The present study aims to find kinematic and/or kinetic patterns of gait characterizing the time course of the disease in 3 different evolutive groups of patients with type II DM: subjects with recently diagnosed type II DM, subjects with overt neuropathy but without clinically evident complications, subjects with diabetic neuropathy and FU.

## Methods

### Study participants

Forty subjects with diagnosis of DM type II were initially recruited from the Diabetic Foot Section of the Dept. of Endocrinology and Metabolism (University Hospital of Pisa, Italy). Eight subjects were excluded because they met one or more exclusion criteria, while two subjects dropped out after preliminary clinical evaluations. The remaining 30 subjects (M/F: 22/8; mean age ± SD: 61,84 ± 10 years) were included in the study.

All participants were divided into three groups according to the stage of evolution of the disease (10 subjects per group): Group 1, including subjects with newly diagnosed DM type II without clinical or instrumental DPN nor FU (group called “DM”); Group 2, with DPN but without FU (group called “DPN”); Group 3, with DPN and a current FU (group called “DNU”) [see Table 1 for detailed description of the groups].

**Table 1:** Characteristics of the groups of patients

	Age (years)	Gender (M/F)	BMI	Diabetes duration (years)
Group 1: DM	63,7 ± 9,4	6/4	27,9 ± 5,2	(Up to 3 months)
Group 2: DPN	62,3 ± 12,9	8/2	28,9 ± 6,1	17,9 ± 10,4
Group 3: DNU	58,5 ± 6,8	8/2	29,5 ± 5	16,8 ± 9,7

*DM* newly diagnosed diabetes, *DPN* diabetes with neuropathy, *DNU* diabetes with neuropathy and ulcer

The three groups were matched for anthropometric variables such as age, weight, height and BMI as confirmed with 1-way ANOVA analysis [age (*P* = 0.64), weight (*P* = 0.29), height (*P* = 0.14) and BMI (*P* = 0.73)].

Inclusion criteria were: diagnosis of DM type II, walking ability for a short distance (10 m) and age over 18 years.

Instead, criteria for exclusion were: severe cardiopulmonary disease, severe lower limbs arthritis, severe motor disability due to other neurological diseases and important cognitive deficits; furthermore, patients with severe foot deformity, current infection of foot ulcer, major amputation (i.e. transmetatarsal amputation or hallux amputation, as well as amputation of more than a toe) or third finger amputation (because of the marker set) were also excluded.

### Gait analysis

All subjects underwent 3-D Gait Analysis performed at Motion Analysis Laboratory of Neurorehabilitation Unit of the University Hospital of Pisa, using the ELITE System (BTS Bioengineering, Milan, Italy).

Before the recordings, general and anthropometric data were collected. Then, spatio-temporal and kinematics data were acquired through six photogrammetric system infrared cameras acquiring at a sampling frequency of 100 Hz. 28 reflective markers were placed on definite anatomical landmarks for kinematic acquisitions, using a modified version of Davis protocol [19]: additional markers were placed at first metatarsophalangeal joint and distal interphalangeal joint of the second toe of both feet, with the aim to better study the foot kinematics.

Kinetic parameters were acquired with two forces plates (Kistler, Switzerland) embedded in the walkway.

Acquisitions were made in standing position and during barefoot walking at self-selected speed, recording at least five trials.

### Data analysis

A first analysis of the data was carried out with BTS Elite Clinic software. Further analysis were performed using MATLAB.

Spatio-temporal parameters calculated were: velocity, cadence, stride time, stride length, step time, step length, step width and the percentages of stance and swing phases compared to the total duration of gait cycle. Furthermore, all temporal parameters were evaluated dividing the gait cycle in sub phases.

In the analysis of the kinematic pattern, a body model accounting for each body segments was used to calculate articular angular excursions along the gait cycle. Several factor were considered: in the sagittal, frontal and transversal plan, the value of the maximum and the minimum angle reached in each joint (hip, knee, ankle and foot) was calculated. Furthermore, the moment of the gait cycle when this maximum or minimum angle is reached was calculated, as well as the dynamic range of motion (ROM) of each joint.

### Statistical analysis

The comparison of the values between groups was analyzed with 1-way analysis of variance (ANOVA). We also investigated group to group differences with Tukey HSD test. The results are described in relation to the significance of *P* < 0,05 and 95 % confidence interval. We calculated effect size by means of eta-squared value to underline the meaningfulness of the differences between groups.

The software analysis used was R (version i386 3.0.2).

## Results

### Effect size

The three groups were recruited in order to be matched. To validate the strength of these results we calculated the effect size for the anthropometric variables (age: 0,975; weight: 0,946; height: 0,996; BMI: 0,969).

The statistical post-hoc analysis showed no significant differences in these variables for any couple of groups.

### Spatio-temporal parameters

Among the spatio-temporal parameters taken into account, a progressive increase of step width mean values with the advance of the disease was evident (Group 1: 225.42 mm ± 27.03; Group 2: 238.80 mm ± 47.7; Group 3: 274.27 mm ± 39.6; P = 0.0265; Eta-squared = 0,2359) with a particularly significant difference comparing Group 1 and 3 (P = 0.0248 at Tukey’s test, 95 % confidence intervals: 5.492098 < μ < 92.19856) [Table 2].

**Table 2:** Step width. Values of step width (mm) of single subjects of the 3 groups, with the mean of the values for each group

	Step width
DM	208.17
DM	251.90
DM	226.43
DM	228.26
DM	267.83
DM	170.12
DM	204.91
DM	222.60
DM	234.00
DM	240.01
Mean ± SD	225.42 ± 27,03
DPN	266.72
DPN	251.67
DPN	268.02
DPN	166.41
DPN	200.57
DPN	278.40
DPN	302.28
DPN	243.35
DPN	250.88
DPN	159.68
Mean ± SD	238.80 ± 47.7
DNU	227.73
DNU	258.78
DNU	295.46
DNU	269.50
DNU	307.63
DNU	247.24
DNU	212.13
DNU	314.00
DNU	272.68
DNU	337.55
Mean ± SD	274.27 ± 39.6

### Kinematic parameters

As regards the maximum value of the angle of internal rotation of the foot, there was a significant difference between the three groups (*P* = 0.0142; Eta-squared = 0,2702); this difference was greater comparing Group 2 and 3 (*P* = 0.0165 at Tukey’s test; 95 % confidence intervals: −25.30359 < μ < −2.2679653). Furthermore, a significant difference between the 3 groups in the minimum value of the angle of abdo-adduction of the hip was found (*P* = 0.0317; Eta-squared = 0,2256); also this difference proved to be greater in the comparison between Group 2 and 3 (*P* = 0.0248 at Tukey’s test, 95 % confidence intervals: −5.449042 < μ < −0.3245327).

**Fig. 1:**
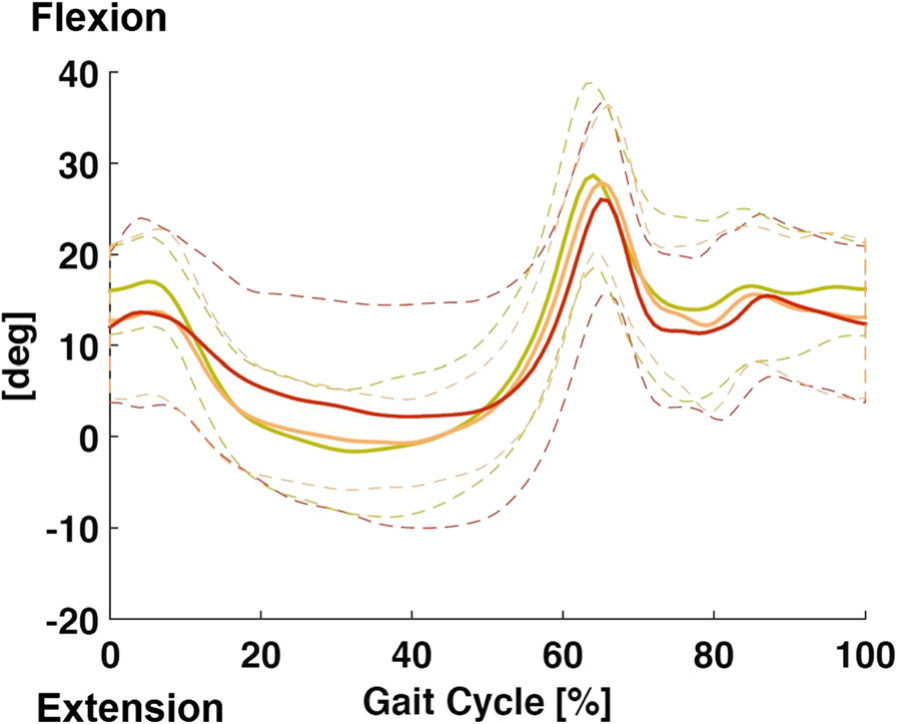
Foot sagittal plane kinematics. Comparison of the metatarso-phalangeal flexion-extension between the three groups, showing a trend of progressive reduction of ROM of flexion-extension with the advancing of the disease

Analyzing ROM in flexion-extension of the metatarso-phalangeal joint, a significant difference between the three groups was observed (*P* = 0.0039; Eta-squared = 0,337), in particular when comparing Group 1 and 3 (*P* = 0.0049 at Tukey’s test, 95 % confidence intervals: −12.483622 < μ < −2.0752676) [Table 3 and Fig. 1].

**Table 3:** Foot sagittal plane kinematics. ROM (°) of flexion-extension of the foot of single subjects of the 3 groups, with the mean of the values

	Flexion-extension
DM	43.62
DM	32.07
DM	39.19
DM	36.32
DM	43.70
DM	46.34
DM	35.36
DM	35.48
DM	36.31
DM	34.75
Mean Group 1	38.31
DPN	42.92
DPN	38.35
DPN	28.96
DPN	34.51
DPN	30.27
DPN	42.66
DPN	35.27
DPN	41.54
DPN	35.81
DPN	40.15
Mean Group 2	37.04
DNU	29.40
DNU	33.11
DNU	25.71
DNU	33.20
DNU	33.69
DNU	36.18
DNU	27.00
DNU	23.19
DNU	35.35
DNU	33.49
Mean Group 3	31.03

### Kinetic parameters

No statistically significant difference in the comparison between groups of the kinetic parameters was found, although it was possible to observe a trend of progressive reduction between groups of the second peak of vertical ground reaction forces (*P* > 0.05; Eta-squared = 0,1089).

## Discussion

As previously mentioned, to date only few research articles studied biomechanical alterations of lower limb in DM patients including subjects with a current or past history of diabetic FU [15–18]. The greater difficulty in exam execution and in data standardization in these patients is certainly a determining factor. We excluded patients with severe foot deformity or major amputations both because of the marker set used and of the increasing between groups comparability. Also, we tried to avoid the contribution exclusively linked to the foot morphology which further alters biomechanics [20].

Our primary finding is that foot segmental kinematics, along with the base of support, differs between our disease groups and might, therefore, be simple and precise parameters to identify patients who will develop an ulcer.

### Spatio-temporal parameters and their effect on biomechanics

No statistically significant differences in spatio-temporal gait parameters like walking speed and cadence resulted from our data analysis. This allows to exclude the intrinsic influence of these factors on the other parameters. In fact, in previous studies Savelberg et al. [21] identified in kinematic and EMG patterns the dependence of each parameter from gait speed (gait-dependent parameters), diabetes (diabetes-dependent parameters) and diabetic polyneuropathy (polyneuropathy-dependent parameters). Removing the dependence from speed, we can assume that the differences found between the groups were definitely linked to the trend of the disease.

### Segmental kinematics of the foot

The first result of our analysis is the absence of significant differences in ROM of the ankle during walking. This result apparently contrasts with the already cited trend of reduction of strength in the dorsal flexion of the ankle. Rao et al. [22] showed that DM subjects had a reduction of passive ROM of ankle dorsiflexion combined with higher stiffness compared to non-diabetic controls at rest. Conversely, while walking at the same speed ankle ROM, stiffness and plantar pressures were not significantly different between DM subjects and controls. In addition to the discrepancy between what measured at rest and what measured during walking, all this seems to suggest a smaller role of the modifications of ankle kinematics in the onset and evolution of FU compared to what is generally expected.

At the same time, we studied intrinsic dynamic ROM of the foot (focusing on metatarsophalangeal joint). Until now, an unbiased understanding of foot kinematics has been difficult to achieve due to the complexity of foot structure and motion [23]. For these reasons, data about segmental kinematics of the foot are less standardized and less reported in literature than other joints. Specific studies reported results useful for clinical interpretation, with different marker sets to study dynamic foot kinematics [24–27], i.e. with particular clinical focus on stance phase, providing information about coronal plane alignment of the rear-foot, transverse and sagittal plane alignment of the metatarsal bones, and changes at the medial longitudinal arch [26]. It was also established the importance of a multisegment foot model for kinematics not to consider the entire foot as a rigid body connected to the shank [28]. Anyway, reproducibility of marker location and skin motion artifact made most of these methods weak [29].

For this aim, we used a modified Davis protocol [19] with additional reflective markers placed at first metatarsophalangeal joint and distal interphalangeal joint of the second toe of both feet. This marker placement protocol was tested in our laboratory in three normal subjects; between-tester repeatability was also assessed. The choice of this protocol was essential to have a simple but useful method to study and analyze more specifically the kinematics of this anatomical district, allowing an objective measurement of a portion of the foot which is important in the pathogenesis of FU. This helped us to find a reduction of metatarsophalangeal flexion-extension ROM, that is more evident in the progressive reduction between groups of the peak of flexion at the end of stance phase [see Fig. 1].

The reduction of ROM in flexion-extension of the metatarsophalangeal joint points out a lower mobility of this anatomical district. Surely these changes play a role in the different load distribution on the foot sole: Rao et al. [11] highlighted that reductions in segmental foot mobility were accompanied by increases in local loading in subjects with DM. The authors suggested that reduction in frontal plane calcaneal mobility during walking may be used as an important functional marker of loss of foot flexibility.

### The wider base of support and the modifications of gait

The results of the present study point out a wider base of support during gait with advancing of the disease, underlining the impaired dynamic control of balance. Patients with DM and DNP have already been described to have gait instability [30]. Unsteadiness may be related to damage in the vestibular, autonomic, and somatic nervous systems [31]; balance impairments reported in people with DM have been related to loss of vestibular function that precedes, in many cases, sensory loss to the feet [32].

As regards the static control of balance, Nardone et al. [33] measured the balance during quiet stance on a stabilometric platform in normal control group and in DNP, Charcot-Marie-Tooth disease type 2 (CMT2) and Charcot-Marie-Tooth disease type 1A (CMT1A) patients. The results showed a larger sway area in DNP and CMT2 patients than in normal subjects or in CMT1A patients (both with eyes open and closed). They postulated that this postural imbalance could be ascribed to a decreased conduction velocity of group II afferent fibres in DNP and CMT2: the somatosensory input from smaller diameter fibres plays an important role in the feedback control both in static and dynamic conditions, involving the stance phase of gait [34].

Anyway, Cavanagh et al. [35] studied DNP patients’ walking on a treadmill suggesting the dominance of efferent input over afferent feedback during gait. Muscle involvement may be secondary to peripheral nerves involvement or a primitive deficit in muscle structure and functioning. The consequence is the reduction of automatic and efficacious postural adaptation through differential muscle contraction [8].

It is important to stress that previous authors investigating gait in diabetic population often correlated the alteration of certain gait parameters with unsteadiness and fall risk [7], but gait parameters taken into consideration were mostly gait speed, step length, time of double support and step-to-step variability. The importance of measuring step width was often underestimated, even if some author [18] already reported a wider base of support in these patients.

### Limitations of the study and other observations

A limitation of the current study could be represented by the small sample size. Using a larger sample sizes may strengthen the statistics of the study.

Another limitation to consider is that recordings were made in a setting of short-medium dimensions. Therefore, the absence of statistically significant differences in gait speed and cadence can be explained because patients walked for distances probably not enough to make out substantial differences in spatio-temporal parameters. It is also important to specify that Gait Analysis was performed at a self-selected speed without any external standardization of walking speed (e.g. using treadmill) in order to investigate actual daily functions of subjects. As regards the long distances, three studies reported that patients with DNP walked slower than healthy controls [21, 28, 36]. However, two studies reported that subjects with DNP walked faster compared to the healthy control group and to diabetic controls [37, 38]. In general, anyway, it is plausible to affirm that with the advance of the disease the walking speed is reduced, also because it is thought that slower speed in neuropathic patients is useful to maintain dynamic stability during overground walking.

Furthermore, the extent of peripheral neuropathy was not investigated. Certainly, monitoring the loss of sensation over time could be a useful adjunct to understand the its contribution to the establishment and maintenance of the phenomenon.

## Conclusions

Our results pointed out two biomechanical factors revealing early modifications of gait in diabetic patients: the reduced excursion in flexion-extension of the foot and the wider base of support. These factors indicate respectively the lower mobility of the foot and the impaired dynamic control of balance with advancing of the disease. Foot segmental kinematics, along with step width, may be proposed as key factors to find a way to intervene in a targeted manner in DM patients before the onset of FU. Prospective longitudinal research is then needed to substantiate or refute this assumption. Anyway, the inclusion of patients with FU in our study allowed us to define an evolutionary path that begins from subjects with newly diagnosed DM type II which does not show any clinical feature of peripheral neuropathy.

The ultimate goal is to help planning more targeted strategies to prevent the occurrence and the recurrence of a FU in diabetic subjects, with an important and significant socio-economic impact.
